# Biomechanical Comparison of Unilateral Biportal Endoscopy and Percutaneous Endoscopic Lumbar Discectomy Using a Finite Element Model

**DOI:** 10.1002/jsp2.70222

**Published:** 2026-10-27

**Authors:** Chang Kyu Lee, Bong Ju Moon, Seong Yi, Yoon Ha, Keung Nyun Kim, Dong Ah Shin

**Affiliations:** ^1^ Department of Neurosurgery, Spine and Spinal Cord Institute Severance Hospital, Yonsei University College of Medicine Seoul Korea; ^2^ Department of Neurosurgery The Spine and Spinal Cord Institute, Gangnam Severance Hospital, Yonsei University College of Medicine Seoul Korea

**Keywords:** finite element analysis, lumbar spine biomechanics, percutaneous endoscopic lumbar discectomy, segmental stability, unilateral biportal endoscopy

## Abstract

**Background:**

Lumbar disc herniation (LDH) remains a significant cause of back and leg pain and disability worldwide, necessitating effective surgical intervention when conservative treatments fail.

**Objective:**

This study aims to compare the biomechanical properties of unilateral biportal endoscopy (UBE) discectomy and percutaneous endoscopic lumbar discectomy (PELD) with those of open microscopic discectomy (OD) for treating LDH.

**Methods:**

Finite element analysis (FEA) was used to evaluate and compare postoperative biomechanical outcomes, focusing on stress distribution, spinal segment flexibility, and postoperative structural integrity. A single patient‐specific L4–L5 finite element model was constructed from a high‐resolution CT dataset and modified to simulate intact, OD, UBE, and PELD conditions.

**Results:**

All three surgical models showed increased range of motion relative to the intact model, with the largest changes in the OD model. The greatest increase occurred in extension (OD, +12.4%; UBE, +4.6%; PELD, +3.5%). Peak von Mises stress in the nucleus pulposus and annulus fibrosus increased across all models, with the greatest increases in OD and the least in PELD. During extension, facet stress increased by 60.0% in the OD model and 14.4% in the UBE model, whereas the PELD model remained essentially unchanged (−1.6%).

**Conclusion:**

Within this single patient‐specific model, models with less posterior osteoligamentous resection showed smaller deviations in range of motion and lower peak stresses. These findings suggest an association between the extent of structural resection and immediate postoperative biomechanical changes, rather than an inherent superiority of any particular technique. The results are model‐specific and hypothesis‐generating, and require validation across multiple patient‐specific models and clinical studies.

## 1 Introduction

Lumbar disc herniation (LDH) is the most common cause of back and leg pain. In most cases, conservative treatment for LDH yields acceptable results. Surgery is considered for patients who have failed conservative treatment and have pain and neurological deficits. A variety of minimally invasive discectomy procedures have been developed as minimally invasive surgery has advanced [1]. Minimally invasive spinal endoscopy is a cutting‐edge surgical technique that has revolutionized the field of spinal surgery. This approach offers several advantages over traditional open surgery, including smaller incisions, reduced trauma to surrounding tissues, faster recovery times, and improved patient outcomes. Kambin introduced percutaneous endoscopic lumbar discectomy (PELD) as a less invasive spinal surgical option in the late 1980s [2, 3]. PELD has been widely used for LDH as a minimally invasive spinal procedure. It has satisfactory outcomes compared with conventional open or microscopic surgery [4, 5].

In recent years, unilateral biportal endoscopy (UBE) has emerged as a rapidly growing surgical method that uses an arthroscopic system to treat LDH. The technique has two independent endoscopic channels, which provide a clear, magnified surgical field, and improve operational flexibility. The anatomical approach and surgical instruments are similar to those used in traditional open microscopic discectomy (OD). Compared with OD, PELD and UBE have good clinical outcomes, with less muscle injury and facet joint preservation [6]. Despite their increasing clinical adoption, the biomechanical consequences of these techniques, particularly in comparison with OD, remain incompletely understood.

Finite element analysis (FEA) provides a well‐established computational framework for evaluating spinal biomechanics and enables quantitative assessment of segmental motion and stress distribution after surgical intervention.

Although OD, UBE, and PELD differ in the extent and location of bony, ligamentous, and discal resection, the immediate biomechanical consequences of these distinct surgical modification patterns remain insufficiently characterized under matched anatomical and loading conditions [6]. Clinical comparisons provide essential outcome data but are confounded by variations in patient anatomy, pathology, surgical indication, and operative technique, making it difficult to assess the biomechanical effects of the surgical modifications under controlled conditions [7]. By applying predefined surgical modifications to the same patient‐specific intact model while maintaining identical material properties and loading conditions, FEA enables a controlled within‐model comparison of their combined biomechanical effects. Therefore, this study aimed to compare changes in segmental range of motion (ROM) and stress distribution among models of simulated OD, UBE, and PELD and to examine whether greater structural preservation was associated with smaller immediate biomechanical alterations.

## 2 Materials and Methods

### 2.1 CT Acquisition and Patient Characteristics

A single patient‐specific L4–L5 finite element model was constructed from computed tomography images of a 24‐year‐old man with no history of lumbar spinal disease, deformity, or prior spinal surgery. The L4–L5 segment showed preserved disc height without osteophytes, endplate sclerosis, facet arthropathy, or radiographic evidence of abnormal bone quality. Axial images were obtained with a slice thickness of 1.0 mm. A radiologically normal segment was selected as a baseline, free of pre‐existing degenerative change, to assess the biomechanical effects of the simulated procedures without confounding by degeneration. No other patient datasets were used. The study was approved by the Institutional Review Board of Yonsei University Severance Hospital (2013‐0515‐001).

### 2.2 Image Segmentation and Three‐Dimensional Reconstruction

The DICOM images were imported into Mimics (Materialise, Leuven, Belgium). Cortical bone, cancellous bone, posterior elements, endplates, facet cartilage, annulus fibrosus, and nucleus pulposus were segmented, and a three‐dimensional surface model of the L4–L5 segment was reconstructed (Figure 1).

**FIGURE 1:**
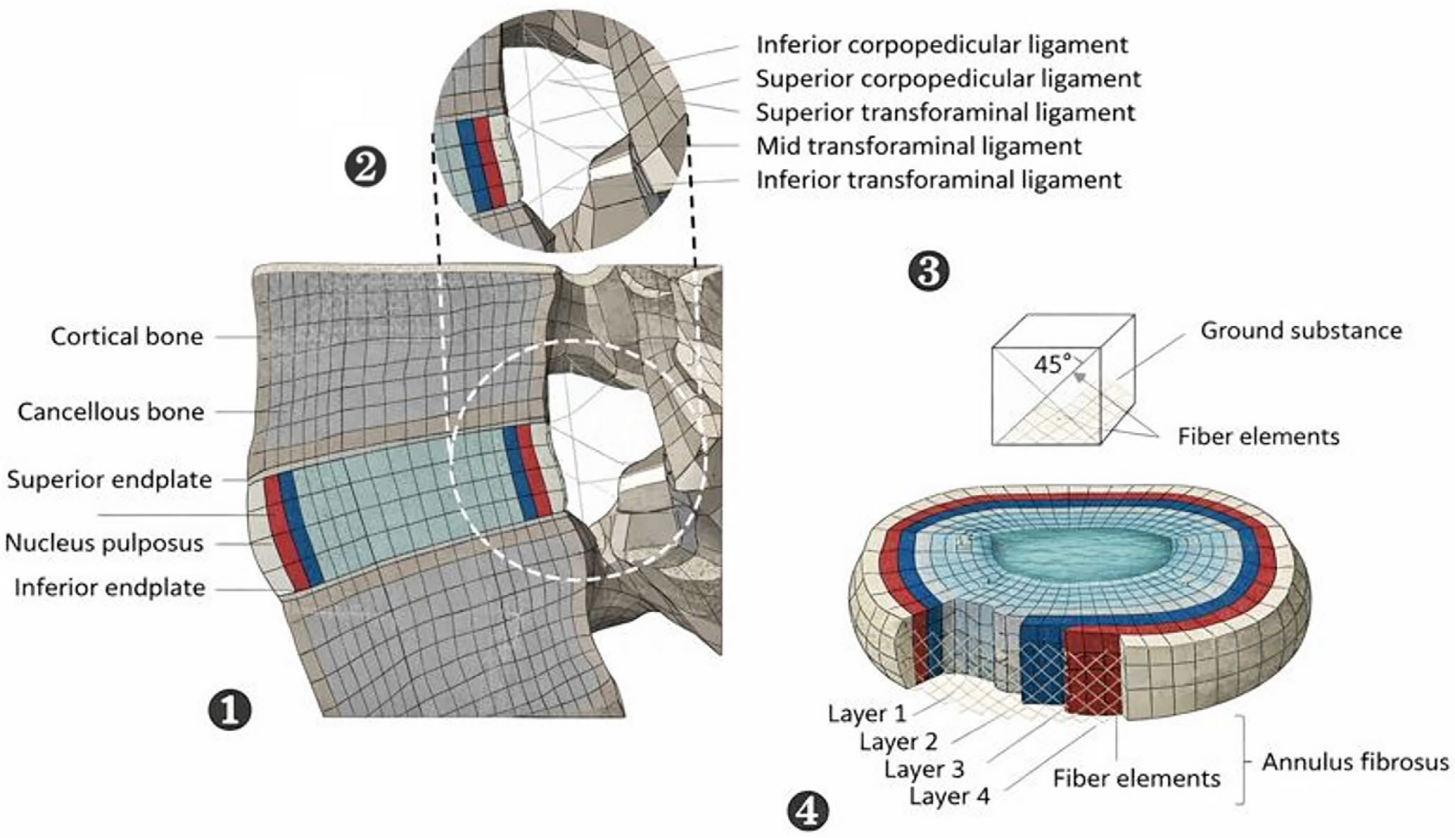
The biomechanical model of the L4–5 segment. (1) A section cut in the midsagittal plane. The model includes the cortical bone, cancellous bone, endplates, and intervertebral disc. (2) In this study, the foraminal ligaments are specifically integrated into the model. (3) The annulus fibrosus is reinforced by fiber elements oriented at 45° to the ground substance. (4) The intervertebral disc has four annular layers.

### 2.3 Mesh Generation

The surface model was converted into a hexahedral mesh using IA‐FEMesh (The University of Iowa, Iowa City, IA). The model was subsequently assembled in Abaqus (Dassault System, Paris, France), in which material properties, interactions, and boundary and loading conditions were assigned.

### 2.4 Material Properties

The vertebral body, intervertebral disc, and cartilage were modeled as linear elastic materials. The vertebral body comprised cortical and cancellous bone, with the posterior elements represented as a unified component. The intervertebral disc consisted of the nucleus pulposus and a four‐layer annulus fibrosus reinforced with fiber elements oriented at ±45° to the ground substance. The spinal ligaments were represented as nonlinear, tension‐only line elements defined by yield deformation and yield stress. Nodes were shared at the interfaces among the nucleus pulposus, annulus fibrosus, endplates, and vertebral bodies. Material properties were adopted from previously validated lumbar finite element models [8] and are summarized in Table 1.

**TABLE 1:** Material properties assigned to the model.

	Young's modulus (N/mm^2^)	Poisson's coefficient	Cross‐section (mm^2^)
Cortical bone	12 000	0.3	
Cancellous bone	100	0.2	
Posterior arch	6000	0.3	
Endplate	300	0.3	
Annulus fibrosus	2.5	0.45	
Nucleus pulposus	1	0.499	
Anterior longitudinal ligament	10		0.5
Posterior longitudinal ligament	20		0.5
Capsular ligament	20		0.6
Ligamentum flavum	50		0.4
Interspinous ligament	3		3
Intertransverse ligament	12		1.8
Supraspinous ligament	3		5
Inferior corpopedicular ligament	50		0.1
Superior corpopedicular ligament	50		0.1
Superior transforaminal ligament	50		0.1
Mid transforaminal ligament	50		0.1
Inferior transforaminal ligament	50		0.1

### 2.5 Validation of the Intact Model

The intact model was validated before any surgical modification. Segmental ROM at L4–L5 during flexion, extension, lateral bending, and axial rotation was measured under a 280 N axial compressive preload combined with a 7.5 N m pure moment, and the results were compared with in vitro data reported by Yamamoto et al. [9]. Because the reference study applied a pure moment without a compressive preload, the two loading protocols differ. Absolute ROM values and the corresponding percentage differences are presented in Table 2. The intact model showed segmental ROM of 6.61° in flexion, 6.52° in extension, 5.12° in lateral bending, and 2.58° in axial rotation. Yamamoto et al. reported corresponding values of 8.9°, 5.8°, 5.7°, and 2.2°, with percentage differences of −25.7%, +12.4%, −10.2%, and +17.3% (Table 2).

**TABLE 2:** Validation of the intact L4–L5 finite element model against published in vitro data.

Motion	Present study 280 N preload +7.5 N m	Yamamoto et al. 10 N m, no preload, *n* = 10	Difference
Flexion	6.61°	8.9°	−25.7%
Extension	6.52°	5.8°	+12.4%
Lateral bending, one side	5.12°	5.7°	−10.2%
Axial rotation, one side	2.58°	2.2°	+17.3%

*Note:* Values represent segmental range of motion at L4–L5; differences are expressed relative to Yamamoto et al. Lateral bending and axial rotation are one‐side values, following the convention of Yamamoto et al.

Yamamoto et al. applied a 10 N m pure moment without preload, whereas the present model applied a 7.5 N m moment with a 280 N axial compressive preload. Both the lower moment and the added preload reduce segmental motion, accounting for the lower flexion value in the present model.

### 2.6 Generation of the Surgical Models

The validated intact model was modified to simulate three surgical conditions: OD, UBE, and PELD. To represent the herniated state, a posterolateral annular defect with an already‐extruded epidural fragment was assumed in the baseline geometry because the fragment lay outside the disc; it was not included in the mesh. The same annular defect was applied to all three surgical models, and identical geometry, material properties, and loading conditions were used throughout.

The simulated procedures were based on published descriptions of each technique. OD was modeled via a posterior interlaminar corridor with partial laminotomy and medial facetectomy [10]. The UBE model included unilateral limited laminotomy with partial flavectomy and preservation of the facet joint [7, 11]. The PELD model represented a transforaminal selective fragmentectomy without foraminoplasty or additional annulotomy, removing only the nucleus pulposus adjacent to the pre‐existing annular tear [2]. Ipsilateral foraminal ligament elements were removed to model the transforaminal corridor [12], which we regard as a modeling assumption rather than an invariable operative step. The facet capsular ligament was preserved in all models. The extent of resection in each model was measured from the finite element mesh and is reported in Table 3.

**TABLE 3:** Quantitative extent of simulated resection in each surgical model.

Parameter	Control (intact)	OD	UBE	PELD
*Osseous structures*				
Lamina volume, mm^3^	2731.60	2328.30	2489.15	2731.60
Lamina resected, mm^3^ (%)^a^	0 (0)	403.30 (29.5)	242.45 (17.8)	0 (0)
Medial facet volume, mm^3^ ^b^	38.54	27.11	38.54	38.54
Medial facet resected, mm^3^ (%)	0 (0)	11.43 (29.7)	0 (0)	0 (0)
*Intervertebral disc*				
Annulus fibrosus volume, mm^3^	7378.26	7173.32	7274.87	7274.87
Annulus resected, mm^3^ (%)^c^	0 (0)	204.94 (2.8)	103.39 (1.4)	103.39 (1.4)
Nucleus pulposus volume, mm^3^	5552.15	5124.13	5441.98	5493.96
Nucleus resected, mm^3^ (%)	0 (0)	428.02 (7.7)	110.17 (2.0)	58.19 (1.0)
*Ligamentous structures*				
Ligamentum flavum^d^	Intact	Removed (ipsilateral)	Partially removed (ipsilateral)	Intact
Facet capsular ligament	Intact	Intact	Intact	Intact
Foraminal ligament	Intact	Intact	Intact	Removed (ipsilateral)

*Note:* All volumes were computed by integrating the element volumes of the corresponding mesh regions before and after resection. The resected proportion was calculated as (*V*_before_ − *V*_after_)/*V*_before_ × 100.

Abbreviations: OD, open microscopic discectomy; PELD, percutaneous endoscopic lumbar discectomy; UBE, unilateral biportal endoscopy.

^a^ Because all three procedures were performed through a unilateral approach, laminar resection is expressed as a percentage of the ipsilateral lamina (1365.80 mm^3^, half of the total laminar volume assuming symmetry). Relative to the total bilateral laminar volume, the resected proportions were 14.8% (OD) and 8.9% (UBE).

^b^ Medial facetectomy was simulated in the OD model only; the facet joint was preserved in the UBE and PELD models.

^c^ In the UBE and PELD models, the annular defect corresponds to the pre‐existing annular tear through which the fragment had extruded, rather than to a surgically created annulotomy; the values are therefore identical. In the OD model, the annulotomy was extended beyond this defect. The extruded epidural fragment itself was not included in the mesh.

^d^ Ligamentous structures were modeled as tension‐only line elements rather than solid volumes, as is conventional in lumbar finite element models; their resection is reported as the extent of elements removed rather than as a volumetric percentage. The facet capsular ligament was preserved in all models.

Osseous and discal resection volumes were obtained by integrating the element volumes of the corresponding mesh regions before and after resection, with the resected proportion calculated as (*V*_before_ − *V*_after_)/*V*_before_ × 100. Because all approaches were unilateral, laminar resection is reported relative to the ipsilateral lamina. Ligamentous structures, being line elements, are reported by the extent of element deletion rather than by volumetric percentage. The quantitative extent of each simulated modification is provided in Table 3 (Figure 2).

**FIGURE 2:**
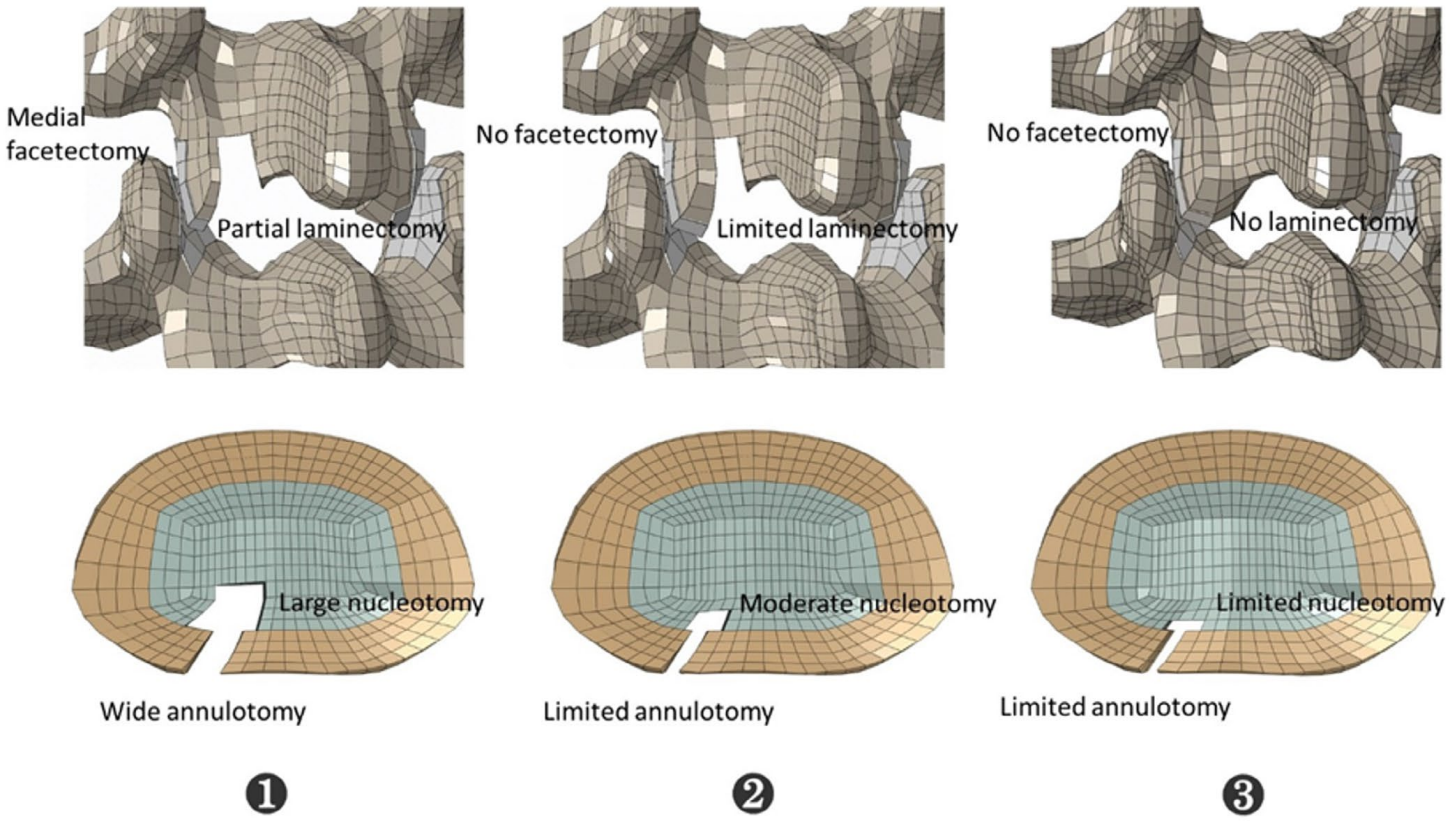
Except for the control group, three surgical models were created. (1) In open microscopic discectomy, medial facetectomy and partial laminectomy were performed. The ligamentum flavum was unilaterally removed. (2) In unilateral biportal endoscopy, a partial laminectomy was performed, but the facet joint was preserved. (3) Percutaneous endoscopic lumbar discectomy did not involve laminectomy. Only the foraminal ligaments were removed, and the least discectomy was performed.

### 2.7 Boundary and Loading Conditions

The inferior endplate of L5 was fully constrained in all 6 degrees of freedom. A reference point at the center of the superior endplate of L4 was coupled to the endplate surface. An axial compressive preload of 280 N was applied along the craniocaudal axis, followed by a pure moment of 7.5 N m applied independently in flexion, extension, lateral bending, and axial rotation. The 280 N preload approximates the axial compressive load acting on the lumbar spine in the upright posture, and the 7.5 N m moment corresponds to the magnitude most widely used for intersegmental ROM evaluation in lumbar finite element studies [9, 13, 14, 15]. Identical boundary and loading conditions were applied to the intact and all surgical models.

### 2.8 Outcome Measures

Segmental ROM was calculated as the relative angular displacement between L4 and L5 under each loading condition; lateral bending and axial rotation are reported as one‐sided values. Maximum von Mises stress was extracted separately from the nucleus pulposus, annulus fibrosus, and facet joints as the maximum nodal value within the corresponding element set, and the corresponding stress distributions were reviewed for each model. Because one deterministic model was analyzed per condition, inferential statistical testing was not performed; results are reported as absolute values and as percentage changes relative to the intact model (Table 4).

**TABLE 4:** Biomechanical outcomes of the intact and surgical models at L4–L5.

Outcome measure	Control (intact)	OD	UBE	PELD
*Range of motion*				
Flexion°	6.61	7.10 (+7.4)	6.87 (+3.9)	6.86 (+3.8)
Extension°	6.52	7.33 (+12.4)	6.82 (+4.6)	6.75 (+3.5)
Lateral bending°	5.12	5.36 (+4.7)	5.27 (+2.9)	5.27 (+2.9)
Axial rotation°	2.58	2.88 (+11.8)	2.68 (+3.9)	2.66 (+3.1)
*Peak von Mises stress*				
Nucleus pulposus, extension, MPa	0.22	0.26 (+18.2)	0.26 (+18.2)	0.24 (+9.1)
Annulus fibrosus, extension, MPa	0.29	0.30 (+3.4)	0.30 (+3.4)	0.29 (0.0)
Nucleus pulposus, flexion, MPa	0.39	0.42 (+7.7)	0.40 (+2.6)	0.40 (+2.6)
Annulus fibrosus, flexion, MPa	0.56	0.59 (+5.4)	0.58 (+3.6)	0.58 (+3.6)
Facet joint, extension, MPa	61.46	98.33 (+60.0)	70.30 (+14.4)	60.49 (−1.6)

*Note:* Values are reported as absolute magnitudes, with percentage changes relative to the intact model shown in parentheses. Negative percentages indicate values lower than those of the intact model. Percentage changes were calculated from unrounded model output. ROM was defined as the relative angular displacement between L4 and L5 under each loading condition. Stress values represent the maximum von Mises stress extracted from the corresponding element set under the indicated loading condition. Lateral bending and axial rotation are reported as one‐sided values. Because each condition was represented by a single deterministic finite element model, measures of dispersion and inferential statistics were not applicable.

Abbreviations: OD, open microscopic discectomy; PELD, transforaminal percutaneous endoscopic lumbar discectomy; UBE, unilateral biportal endoscopy.

## 3 Results

### 3.1 The Range of Motion (ROM)

All three surgical models showed increased segmental ROM relative to the intact model, with the largest increases in the OD model and values closer to the intact condition in the UBE and PELD models (Figure 3, Table 4). The greatest change occurred in extension, with ROM increasing by 12.4% in the OD model, compared with 4.6% in UBE and 3.5% in PELD. Flexion and axial rotation followed the same pattern, with increases of 7.4% and 11.8% in the OD model and 3.1% to 3.9% in the endoscopic models. Lateral bending was the least affected motion, with increases of 4.7% or less in all models.

**FIGURE 3:**
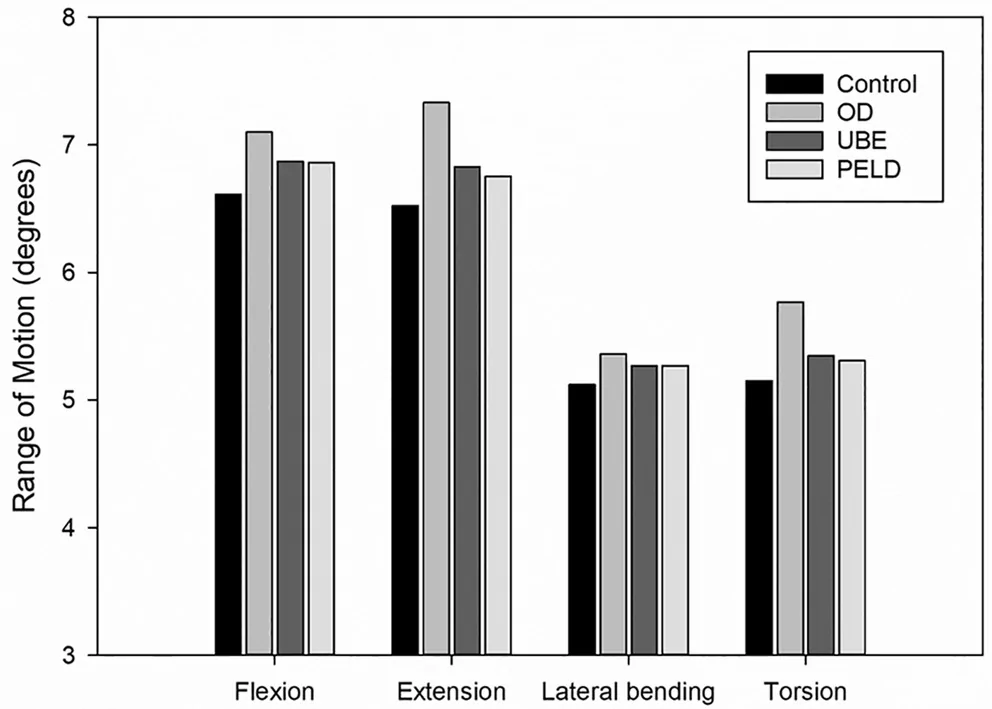
Comparison of range of motion (ROM) under different loading conditions.

### 3.2 The Maximum von Mises Stress on the Intervertebral Disc

During extension, peak von Mises stress in the nucleus pulposus increased across all surgical models, with the greatest increases in the OD and UBE models (0.26 MPa, +18.2%) and the smallest in the PELD model (0.24 MPa, +9.1%) (Figure 4, Table 4). Annular stress followed the same pattern, reaching 0.30 MPa in the OD and UBE models (+3.4%), whereas the PELD model remained at the intact value of 0.29 MPa (Figure 5).

**FIGURE 4:**
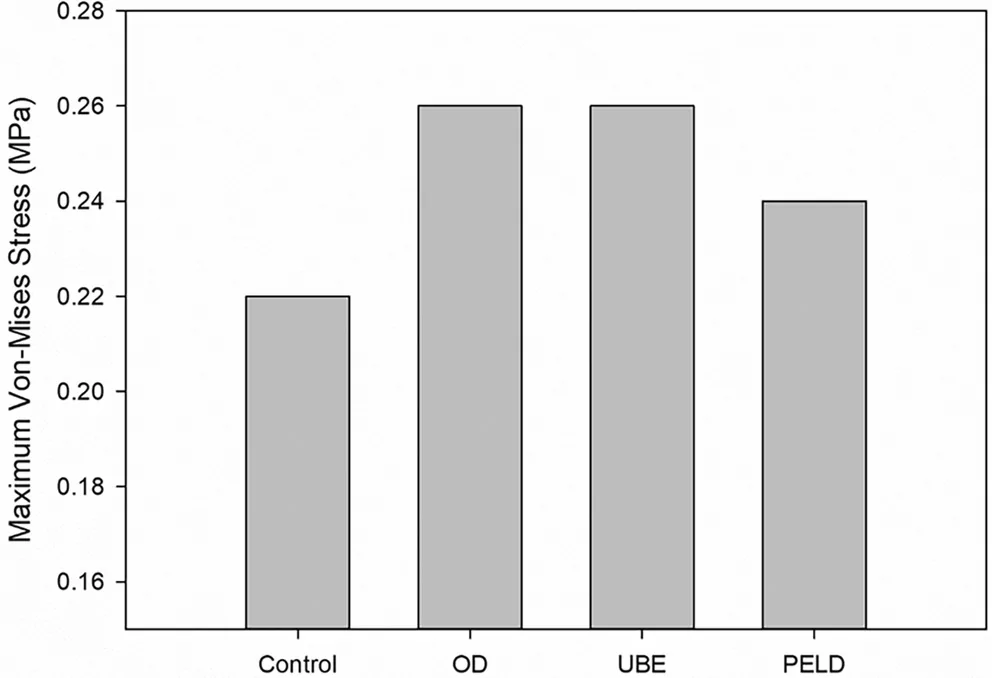
The maximum von Mises stress on the nucleus pulposus during extension.

**FIGURE 5:**
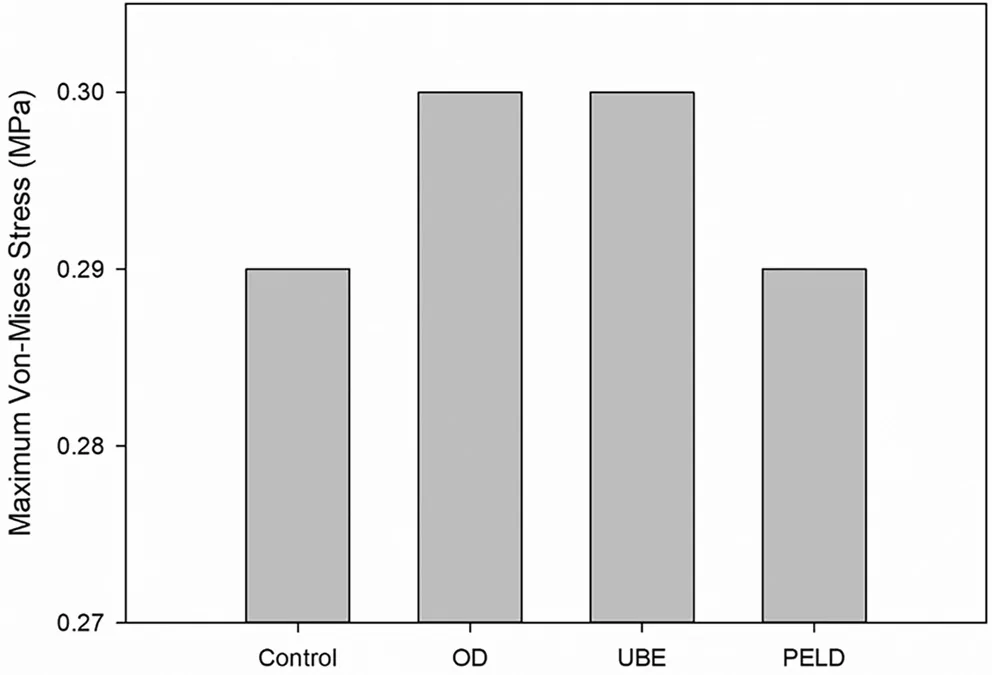
The maximum von Mises stress on the annulus fibrosus during extension.

During flexion, nuclear stress peaked in the OD model (0.42 MPa, +7.7%), with the UBE and PELD models both at 0.40 MPa (+2.6%) (Figure 6). Annular stress reached 0.59 MPa in the OD model and 0.58 MPa in the UBE and PELD models, compared with 0.56 MPa in the intact model (Figure 7).

**FIGURE 6:**
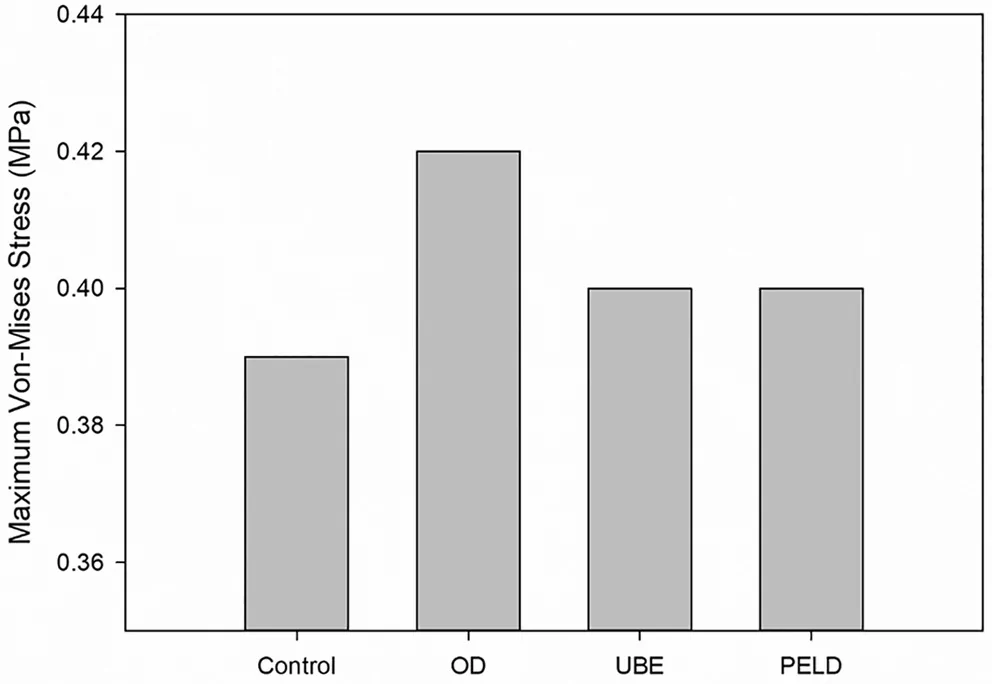
The maximum von Mises stress on the nucleus pulposus during flexion.

**FIGURE 7:**
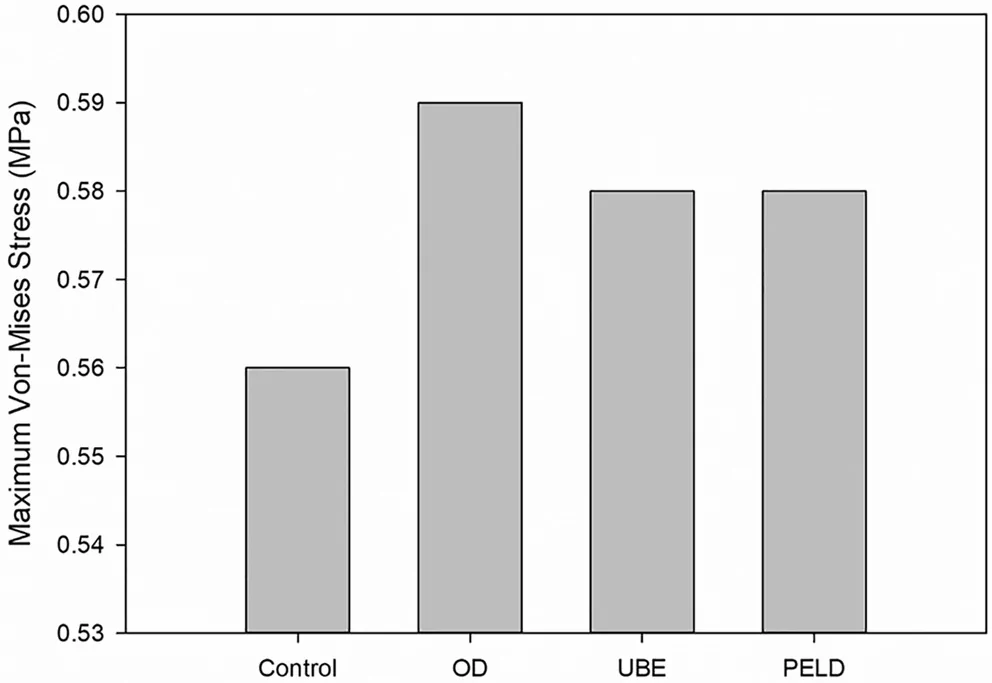
The maximum von Mises stress on the annulus fibrosus during flexion.

### 3.3 The Maximum von Mises Stress on the Facet Joints

Facet stress during flexion was low across all models. During extension, peak facet stress was 61.46 MPa in the intact model, increased to 98.33 MPa in the OD model (+60.0%) and 70.30 MPa in the UBE model (+14.4%), whereas the PELD model remained essentially unchanged at 60.49 MPa (−1.6%) (Figure 8, Table 4). In the OD model, the stress concentration was most pronounced in the superior portion of the L5 facet, whereas the UBE model showed a more heterogeneous distribution.

**FIGURE 8:**
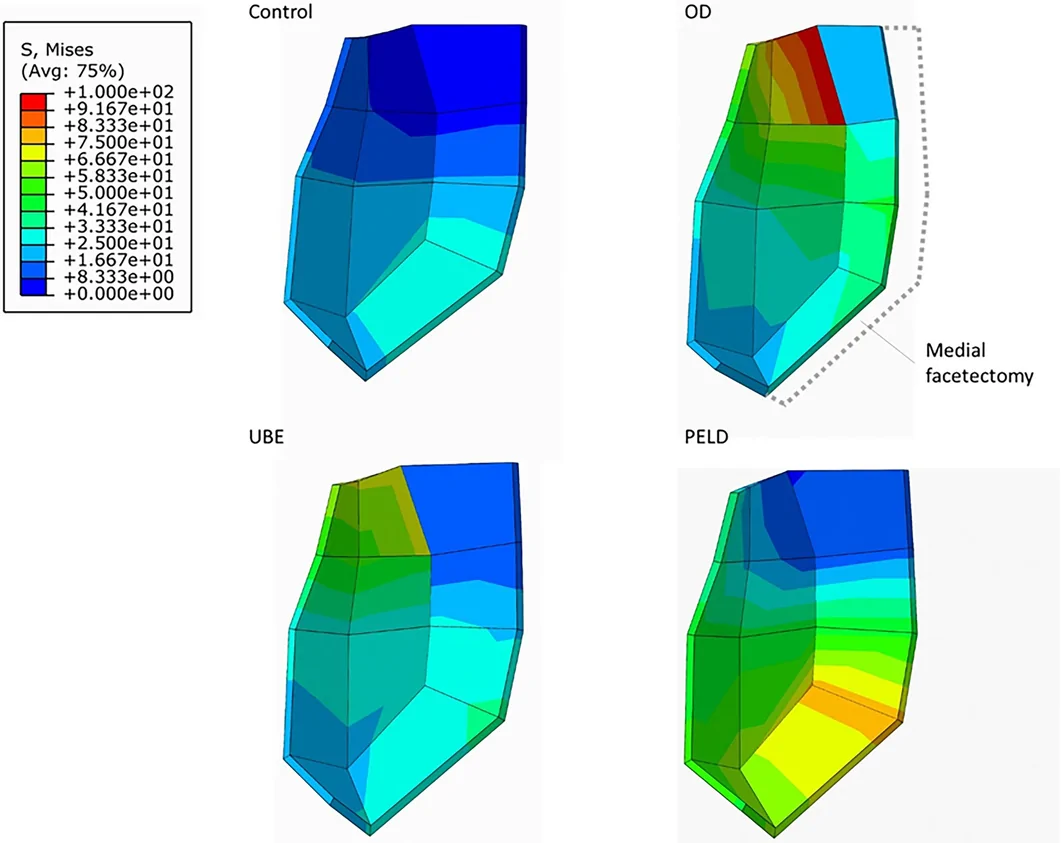
The maximum von Mises stress on the facet joint during extension.

## 4 Discussion

In this patient‐specific finite element model, the UBE and PELD models showed smaller deviations from the intact condition than the OD model. Compared with OD, both endoscopic models were associated with smaller increases in ROM and lower peak stresses in the nucleus pulposus, annulus fibrosus, and facet joints, whereas the OD model showed the largest changes, particularly during extension. These patterns are consistent with the view that more extensive tissue resection is accompanied by greater alteration of segmental mechanics [16].

The quantitative resection data indicate that these differences were driven mainly by the extent of posterior osseous resection rather than by the surgical corridor itself. Discal resection was proportionally limited in all models (annulus, 1.4%–2.8%; nucleus pulposus, 1.0%–7.7%), whereas the OD model involved resection of 29.5% of the ipsilateral lamina and 29.7% of the modeled medial facet region, the UBE model 17.8% of the lamina with preservation of the facet joint, and the PELD model neither laminotomy nor facetectomy. Cadaveric and computational studies have consistently shown that resection of the posterior osseous structures increases segmental ROM and alters load transmission, with the magnitude of the change scaling with the extent of resection [6, 17]. Our findings are consistent with this relationship, and the graded pattern observed across the three models supports the value of selective and minimal resection. The PELD model in the present study represented a facet‐preserving transforaminal fragmentectomy without foraminotomy, foraminoplasty, or facetectomy. Therefore, the minimal change in facet stress observed in this model is most applicable to cases in which facet resection is not required. In foraminal or far‐lateral disc herniations requiring foraminotomy or partial facetectomy, greater changes in facet loading and segmental mechanics may occur.

These biomechanical patterns may help explain the clinical outcomes reported for tissue‐preserving endoscopic procedures [7]. Both UBE and PELD have shown favorable results in postoperative pain, functional recovery, and complication rates [5, 18], and finite element studies have reported that both techniques maintain near‐physiological loading patterns in normal and osteoporotic spines [19]. Meta‐analyses suggest that the two approaches yield comparable improvements in pain and disability scores, with PELD associated with shorter operative time, reduced bleeding, and earlier discharge [4, 5, 20], whereas UBE has been reported to provide a wider working corridor and greater instrument maneuverability at the cost of a longer learning curve [18, 21]. The present findings offer a possible mechanical basis for these observations, as procedures that preserve more of the posterior osteoligamentous complex produce segmental behavior closest to the intact state. These relationships are mechanistic interpretations of a single model rather than direct clinical evidence, and the clinical relevance of differences of this magnitude remains to be established.

A strength of this study is the use of patient‐derived CT data to construct a model that reproduces the anatomy of an individual segment [22], allowing the three procedures to be compared under identical geometry, material properties, and loading conditions. This design isolates the effect of the simulated resection patterns in a way that clinical comparisons cannot, because the latter are influenced by variations in patient anatomy, pathology, surgical indication, and operative technique. FEA nonetheless has intrinsic limitations: it does not incorporate biological healing, soft‐tissue dynamics, or neuromuscular control and is typically based on idealized geometry [16]. Extrapolation to clinical practice should therefore be made with caution, and further validation using cadaveric and prospective clinical studies is required.

Patient‐specific finite element modeling may be integrated into preoperative planning to estimate the biomechanical consequences of a given resection pattern. Studies using multiple subject‐specific models and dynamic simulations would help clarify whether the structure–function relationship observed here translates into differences in long‐term clinical outcome [19].

Several limitations should be acknowledged. First, the model was reconstructed from a single L4–L5 segment of one patient. A radiologically normal segment served as the baseline, and a standardized annular defect was introduced rather than derived from a specific pathological case. The extruded epidural fragment was not explicitly included in the finite element mesh. Therefore, the findings may not be generalizable to other lumbar levels, different anatomical configurations, or varying degrees of disc degeneration.

Second, each surgical model incorporated a distinct combination of bony, ligamentous, and discal modifications, so the independent biomechanical contribution of each structure could not be isolated. In addition, the facet capsular ligament was preserved in all models. Potential capsular injury associated with OD was therefore not accounted for, which may have led to an underestimation of the biomechanical alteration in the OD model.

Third, only the immediate postoperative response was evaluated. Tissue healing, postoperative remodeling, recurrent herniation, and progressive degeneration were not modeled. Therefore, the results should be interpreted as immediate mechanical consequences rather than long‐term clinical outcomes.

Finally, sensitivity analyses of material properties, ligament stiffness, and loading conditions were not performed, and muscle forces and dynamic loading were not incorporated. These factors may affect the absolute magnitude of the calculated parameters. However, all surgical models were derived from the same validated intact model using identical material properties, mesh characteristics, and loading conditions, enabling internally consistent within‐model comparisons. Future studies incorporating multiple subject‐specific models, sensitivity analyses, and physiological muscle forces are needed to improve generalizability and clinical applicability.

## 5 Conclusion

Within a single patient‐specific finite element model, models with less posterior osteoligamentous resection typically showed smaller deviations in segmental ROM and smaller changes in certain disc and facet joint stress measures. These results suggest that the degree of structural preservation, rather than the surgical approach itself, might substantially influence immediate postoperative biomechanical behavior. However, these findings are specific to the model used and are hypothesis‐generating, requiring further validation through multiple anatomical models, sensitivity analyses, and clinical or experimental correlation.

## Author Contributions

Conceptualization: Chang Kyu Lee, Dong Ah Shin. Methodology: Chang Kyu Lee, Dong Ah Shin. Finite element modeling and formal analysis: Dong Ah Shin. Data interpretation: Chang Kyu Lee, Bong Ju Moon, Dong Ah Shin. Writing – original draft: Chang Kyu Lee. Writing – review and editing: Bong Ju Moon, Seong Yi, Yoon Ha, Keung Nyun Kim, Dong Ah Shin. Supervision: Dong Ah Shin.

## Conflicts of Interest

The authors declare no conflicts of interest.

## Data Availability

The data that support the findings of this study are available on request from the corresponding author. The data are not publicly available due to privacy or ethical restrictions.
